# Runx Transcription Factors Repress Human and Murine c-Myc Expression in a DNA-Binding and C-Terminally Dependent Manner

**DOI:** 10.1371/journal.pone.0069083

**Published:** 2013-07-18

**Authors:** Paejonette T. Jacobs, Li Cao, Jeremy B. Samon, Christyne A. Kane, Emmett E. Hedblom, Anne Bowcock, Janice C. Telfer

**Affiliations:** 1 Program in Molecular and Cellular Biology, University of Massachusetts Amherst, Amherst, Massachusetts, United States of America; 2 Department of Genetics, Pediatrics and Medicine, Washington University School of Medicine, St. Louis, Missouri, United States of America; 3 Quntiles, Medical Education Department, Hawthorne, New York, United States of America; 4 Department of Veterinary and Animal Sciences, University of Massachusetts Amherst, Amherst, Massachusetts, United States of America; Indian Institute of Science, India

## Abstract

The transcription factors Runx1 and c-Myc have individually been shown to regulate important gene targets as well as to collaborate in oncogenesis. However, it is unknown whether there is a regulatory relationship between the two genes. In this study, we investigated the transcriptional regulation of endogenous *c-Myc* by Runx1 in the human T cell line Jurkat and murine primary hematopoietic cells. Endogenous Runx1 binds to multiple sites in the *c-Myc* locus upstream of the *c-Myc* transcriptional start site. Cells transduced with a C-terminally truncated Runx1 (Runx1.d190), which lacks important cofactor interaction sites and can block C-terminal-dependent functions of all Runx transcription factors, showed increased transcription of *c-Myc*. In order to monitor c-Myc expression in response to early and transiently-acting Runx1.d190, we generated a cell membrane-permeable TAT-Runx1.d190 fusion protein. Murine splenocytes treated with TAT-Runx1.d190 showed an increase in the transcription of *c-Myc* within 2 hours, peaking at 4 hours post-treatment and declining thereafter. This effect is dependent on the ability of Runx1.d190 to bind to DNA. The increase in *c-Myc* transcripts is correlated with increased c-Myc protein levels. Collectively, these data show that Runx1 directly regulates *c-Myc* transcription in a C-terminal- and DNA-binding-dependent manner.

## Introduction

The transcription factor Runx1 (also known as AML-1, PEPB2αB, CBFα2) is a member of the Runx family of transcription factors. It was originally isolated as a regulator of viral enhancers [1,2] as well as the target of chromosomal translocations in human leukemia [3,4], and plays critical roles in hematopoiesis [5–12]. There are three mammalian Runx family members, which share a highly conserved Runt homology domain that is responsible for binding to DNA and was first characterized based on significant homology to the Drosophila pair-rule gene *runt* [1,3,13–19]. Besides the highly conserved runt domain, Runx family members also contain a nuclear localization sequence (NLS) C-terminally juxtaposed to the runt domain as well as a downstream nuclear matrix targeting sequence (NMTS) [20,21]. The C-terminus of Runx transcription factors also contain sites for cofactors so that Runx transcription factors nucleate complexes that enhance or repress transcription in a cell context- or gene locus-dependent manner [20,22–34]. The deletion of the Runx1 C-terminus downstream of the NLS (Runx1.d190) increases DNA-binding affinity and blocks Runx activity that is dependent on the presence of the Runx C-terminus, such as *Foxp3* transactivation and CD4 silencing [35–37]. CD4 can be silenced by all three Runx family members; Runx1.d190 blocks all CD4-silencing activity [36]. RAG-1 and RAG-2 are also silenced by an intergenic silencer whose activity is dependent on one Runx binding site [38].

The interaction of Runx1 with its ubiquitously expressed non-DNA-binding partner CBFβ via the Runt domain increases the affinity of Runx1 for DNA [39–42]. Moreover, the binding of CBFβ to Runx1 decreases degradation of Runx1 [43]. Runx1 binds to the DNA consensus sequence YGYGGT, where Y represents a pyrimidine [15,39,44]. Single missense mutations within the Runt domain severely diminish or even ablate binding Runx1 DNA binding and are associated with myelodysplasia and the development of acute myeloid leukemia, perhaps by haploinsufficiency and lowered gene dosage or by interference with the homodimerization of wildtype Runx1 or its binding to CBFβ. The translocation product AML1-ETO and frameshift and nonsense mutations resulting in the loss of the C-terminus, with concurrent retention of nuclear localization, CBFβ-binding and DNA-binding, are thought to be associated with leukemia through dominant opposition to C-terminally-dependent functions of endogenous Runx transcription factors [45–50].

The master regulatory transcription factor c-Myc also is critical for hematopoiesis and oncogenesis, and regulates the transcription of 15% of all genes (www.myc-cancer-gene.org [51–53]). C-Myc controls various fundamental and diametrically opposed cellular processes such as cell division, growth, differentiation and apoptosis in a cell context-dependent manner [54–57]. It is commonly elevated in aggressive cancer cells, accumulating at the promoters of actively transcribed genes and amplifying the cancer cell transcriptome [58]. Transient expression of c-Myc is also important for the cellular reprogramming that generates induced pluripotent stem cells [59,60]. C-Myc is regulated at transcriptional and translational levels primarily by mitogenic signals associated with growth and proliferation [61,62]. C-Myc protein is also strictly regulated and has a very short half-life of less than 30 minutes, further highlighting the importance of the tight regulation of the gene and its protein product in a normal cell [63–65].

Runx1 cooperates with c-Myc in oncogenesis as well as in accelerating the development of Myc-induced lymphomas [66–68]. However, it is unknown whether there is a direct regulatory relationship between the two genes. In this study we investigate whether Runx1 directly regulates the expression of c-Myc in hematopoietic cells. We show that endogenous Runx1, as well as a C-terminally truncated form of Runx1 (Runx1.d190), directly bind to both the human and murine *c-Myc* loci. We also show that introduction of Runx1.d190 increases the transcription and protein levels of c-Myc in immune system cells. Since Runx1.d190 blocks functions of Runx transcription factors that require association of the Runx C-terminus with Runx-binding sites on the DNA, it is likely that full-length Runx1 represses *c-Myc* transactivation.

## Materials and Methods

### Ethical statement

All animal work was conducted in accordance with an animal use protocol approved by the University of Massachusetts Amherst Institutional Animal Care and Use Committee (2011-0046).

### Plasmid constructs

The DNA sequences corresponding to the first 204 amino acids of distal Runx1 (distal Runx1.d190) and full-length distal Runx1 (Runx1 FL) were subcloned from MSCV constructs [36] by digestion with BglII and EcoRI and ligation into the BglII/EcoRI-digested vector pEGFP-N1 (Clontech, Mountain View, CA), in frame at its 3’ end with EGFP. The DNA sequence corresponding to the first 204 amino acids of distal Runx1 (distal Runx1.d190) was subcloned into the EcoRI-NheI sites of the pLEIGW [69] lentiviral vector. Distal Runx1.d190 was PCR-amplified with primers containing EcoRI and XhoI sites: forward 5’-GAGGAATTCGATGGCTTCAGACAGCATTTTTGAGTCATTTCC-3’, reverse 5’-CGGCTCGAGCCCGGGCTTGGTCTGATCATCTAG-3’. The DNA was ligated into the pET30 vector (EMD Millipore, Billerica, MA) or the pET 28b TAT vector 2.1 (generous gift of Dr. S.F. Dowdy) and confirmed by sequencing. The QuikChange Site-Directed Mutagenesis kit (Agilent Technologies, Santa Clara, CA) was used to generate the lysine 167 to alanine (K167A) mutation in the TAT-distal Runx1.d190 construct according to the manufacturer’s instructions and using the primers: forward, 5’-CCATAGAGCCATCGCAATCACAGTGGACGGCCCC-3’ and reverse, 5’-GGGGCCGTCCACTGTGATTGCGATGG CTCTATG-3’. The construct was sequenced to confirm the presence of the lysine 167 to alanine mutation.

### Transfection and staining of 293T cells

293T cells were transfected with 6 µL of FuGene HD (Roche, Indianapolis, IN) and 1 µg of pEGFP-N1 vector alone, or pEGFP-N1 with Runx1.d190 or full-length Runx1 fused in frame to the 5’ end of EGFP as per manufacturer’s instructions. Forty-eight hours after transfection, cells were stained with 1 µg/mL Hoescht 33342 (Pierce, Rockford, IL) in PBS. Images were acquired with an Axiovert 200M Zeiss microscope, 40x, and Improvision Openlab software.

### Lentiviral packaging and infection

Supernatants were harvested 48 hours after transfection of 293T cells with 1 µg of the pLEIGW-Runx1.d190 construct or empty pLEIGW and 6 µL of FuGene 6 (Roche). Lentiviral supernatants along with 6 µL FuGene 6 were added to 2×10^5^ Jurkat T cells (clone E6-1, American Type Culture Collection, Manassas, VA, USA) that had been seeded into six-well plates 16 hours previously. The cells were left to recover for 48 hours after which they were analyzed by flow cytometry. GFP^+^ cells were enriched to more than 90% on a cell sorter and maintained in RPMI 1640 (Invitrogen, Carlsbad, CA) with 10% heat-inactivated FBS and antibiotics at 37°C and 5% CO_2_.

### Microarray data analysis

Jurkat T cells transduced with empty vector pLEIGW or pLEIGW-Runx1.d190, cloned and matched for comparable EGFP expression were lysed in Qiazol (Qiagen). RNA was extracted according to the manufacturer’s instructions and was run on 2 Illumina Mouse6 v2 BeadArrays to provide a technical replicate for each experiment. Raw expression data were analyzed in R (v 2.10.1) using the Bioconductor (v 2.6.1) beadarray package (v 1.14.0) [70–72]. Limma (v 3.2.3) was used to log_2_ transform and quantile normalize raw expression values [73]. For differential expression analysis arrays were classified either as “Control” or “Experimental”. Replicate arrays were treated as true technical replicates and not biological replicates; correlation between technical replicates was calculated using the “duplicateCorrelation” limma function. The technical replicate correlation was used during the initial “lmFit” linear modeling step. The probability of a true difference in expression (empirical Bayes statistics) between classes was calculated using the “eBayes” function. The probability of differential expression was corrected for multiple tests by the false discovery rate (FDR; Benjamini and Hochberg method) [74]. Probes with p-values less than 0.05 were considered to be differentially expressed. Of the 45,281 transcript probes interrogated by the Illumina array a total of 7,034 probes (15.5%) met our cutoff criteria.

### Chromatin immunoprecipitation (ChIP) assay

The ChIP assays were carried out using Protein G agarose beads (Millipore, Billerica, MA) according to manufacturer’s instructions, with the following modifications. 2 x 10^6^ cells/mL were fixed with 1% formaldehyde (methanol-free, Ted Pella, Redding, CA) for 10 minutes at 37^o^C. To prepare chromatin, cells were sonicated at a concentration of 2 x 10^6^ cells/300 µL. Jurkat T cells were sonicated 3 times on ice at 25% power for 10 second pulses and splenocytes were sonicated for 3 times on ice at 30% power for 10 second pulses. Chromatin was immunoprecipitated with the control antibody rabbit pre-immune sera, rabbit polyclonal murine anti-distal Runx1 [37], IgG (Southern Biotech, Birmingham, AL) or a His-probe (AD 1.1.10) antibody (Santa Cruz Biotechnology, Santa Cruz, CA). Protein-DNA complexes were eluted by incubating tubes at 50^o^C for 10 minutes then at room temperature for 10 minutes with rotation for a total of 2 times. DNA was purified using QIAEX II resin (Qiagen). 1-2 µL of the DNA was used as a template in a 25 µL reaction using PCR master mix or Hot Start PCR master mix (Promega) according to the manufacturer’s instructions. Murine *c-Myc* primers and conditions were as follows: (i) -0.9 kb forward 5’-AGGGTACATGGCGTATTGTGTGGA-3’, -0.9 kb reverse 5’-ATGAATTAACTGCGCGCCCGA-3’; (ii) -4.25 kb forward 5’-GGGTACAGTACGGGCAAGTC-3’, -4.25 kb reverse 5’-TGGGTAGAGCTGACCCTCAA-3’; (iii) -5.37 kb forward 5’-AAGCGTCTCAAGGATGACCGTTC-3’, -5.37 kb reverse 5’-AACAGGGCCTCATTTGTGGTCA-3’; and (iv) -7.63 kb forward 5’-CCATATCTGCACACTGAAGCA-3’, -7.63 kb reverse 5’-TGGGTCTCCTGATGTTCCTC- 3’. Cycling conditions: 95^°^C, 2 minutes; 95^°^C, 30 seconds; 54^°^C (primer set iv) or 58^°^C (primer sets i, ii and iii), 30 seconds; 72^°^C, 30 seconds for 35 cycles. Human *c-Myc* primers and conditions were as follows: (i) -0.83 kb forward, 5’-CACTCTCCCTGGGACTCTTG-3’, -0.83 kb reverse 5’-CAGCCGAGCACTCTAGCTCT-3’; (ii) -7.86 kb forward 5’-AAGGAGGCCTTTCTCTGACAGCTA-3’, -7.86 kb reverse 5’-CTCAGCACTTTGGTTCAGGCAGTT-3’; and (iii) -8.93 kb forward 5’-AATGCCCAGATCCACTCACCAAGA-3’ -8.93 kb reverse 5’-TTGGAGACAATTCCAAACCCACCC-3’. Cycling conditions: 95^°^C, 30 seconds; 56^°^C (primer set i), 59^°^C (primer sets ii and iii), 30 seconds; 72 ^°^C, 30 seconds for 29-35 cycles. Adobe Photoshop (San Jose, CA) was used to quantify band intensity and background.

### Anti-distal Runx1 antibody affinity comparison

2.5 µg of histidine-tagged Runx1.d190 or TAT-Runx1.d190 was incubated with 10 µL of 50% slurry Nickel Sepharose 6 Fast Flow beads (GE Healthcare, Piscataway, NJ) in 100 µL of PBS containing 1 µg/mL pepstatin and 1 µg/mL aprotinin at 4^o^C for 3 hours. Samples were made up to 1 mL volumes with PBS containing 1 µg/mL pepstatin and 1 µg/mL aprotinin and fixed with 1% formaldehyde (methanol-free, Ted Pella) at 37^o^C for 10 minutes to mimic ChIP conditions or samples were left unfixed for later assessment of protein retention by the beads. The supernatant was removed and the beads were washed with ice-cold PBS containing 1 µg/mL pepstatin and 1 µg/mL aprotinin. The beads were resuspended into 1 mL of ChIP dilution buffer containing 1 µg/mL pepstatin and 1 µg/mL aprotinin. Anti-mouse distal Runx1 or preimmune sera was added after which the mixture was incubated overnight at 4^o^C. The beads were subsequently washed under ChIP conditions. 10 µL of 2x Laemmli buffer was added to the samples, which were then boiled at 95^o^C for 10 minutes. The entire sample was loaded onto a 12% SDS-PAGE gel. Immunoblot analysis was carried out as described below to probe for bound anti-distal Runx1 immunoglobulin on fixed protein beads (anti-rabbit IgG-HRP, Santa Cruz Biotechnology) and bound polyhistidine-tagged protein on unfixed beads (Tetra-His antibody from Qiagen, Valencia, CA).

### Purification of and transduction with TAT-Runx1.d190 and TAT-Runx1.d190-KI67A protein

Bacterial cultures containing TAT-distal Runx1.d190 or TAT-distal Runx1.d190-K167A inserted into pET 28b TAT 2.1 plasmid were grown in Terrific broth at 37^o^C with shaking and diluted 1:10 after 16 hours. 500 µM IPTG was added and the culture was shaken for 5 hours at 37^o^C. Cells were centrifuged at 5000 rpm for 10 minutes, resuspended in 10 mL of buffer Z (8M urea, 100mM NaCl, 20 mM HEPES pH 8.0) and sonicated 3 times on ice at 30% for 15 second pulses. The lysate was clarified by centrifugation at 12,000 rpm for 10 minutes at 4^o^C. Halt protease inhibitor cocktail (Thermo Scientific, Rockford, IL) was added (according to manufacturer’s recommendations) as well as 20mM imidazole. The lysate was incubated with 5 mL 50% slurry Nickel Sepharose 6 Fast Flow beads (GE Healthcare) in buffer Z plus 20 mM imidazole overnight at 4^o^C. The beads were washed with 5 bead volumes of 20 mM imidazole in buffer Z for 15 minutes at 4^o^C for a total of 3 times. The protein was eluted by adding 2 bead volumes of 200 mM imidazole in buffer Z and incubating for 2 hours at 4^o^C with rotation. The beads were collected at 500 x g for 5 minutes at 4^o^C and the elution was repeated using 500 mM imidazole in buffer Z with rotation overnight at 4^o^C. The combined protein fractions was diluted with one volume of 20 mM HEPES pH 8.0 and filtered after which the protein was concentrated using Amicon Ultra-10K MWCO (Millipore) according to the manufacturer’s instructions. The urea/imidazole buffer was further exchanged for PBS, pH 7.4 containing 10% glycerol, 1 µg/mL pepstatin and 1 µg/mL aprotinin using a PD-10 (GE Healthcare) desalting column according to manufacturer’s instructions. LPS was removed by adding 60 µL polymyxin bead suspension (Sigma, St. Louis, MO) per milliliter of eluted protein and incubating with rotation for 5 minutes at 4^o^C. The purified protein was centrifuged at 13,000 rpm for 10 minutes after which it was filtered to 0.2 microns, aliquoted, flash frozen and stored at -80^o^C. The protein concentration was determined by comparison to BSA standards on a Coomassie stained SDS-PAGE gel.

Red blood cells in C57Bl/6J splenocytes were lysed by adding 0.9 mL lysis buffer (144 mM ammonium chloride, 17 mM Tris-HCl pH 7.2), incubating for 5 minutes, after which the cells were washed twice with CBE (PBS, 0.2% BSA and 1mM EDTA, pH 7.4). The cells were resuspended in HL1 serum free media (Lonza, Allendale, NJ) supplemented with 2mM Glutamax, 50 units/mL penicillin, 50 µg/mL streptomycin and 0.05 µg/mL gentamycin (Invitrogen). 6-8 x 10^6^ cells were treated with 0.5 µM TAT peptide 48-57 (Anaspec, Fremont, CA) or TAT-distal Runx1.d190 for 1-4 hours in a 1 mL volume.

### Immunoblot analysis

For analysis of TAT fusion protein purification, proteins were separated on a 12% SDS-PAGE gel and transferred to Immobilon P (Millipore). The blot was probed with Tetra-His antibody (Qiagen) according to manufacturer’s instructions.

For analysis of c-Myc protein levels, splenocytes were harvested from C57Bl/6J mice and the red blood cells were lysed as described above. 6-8 x 10^6^ cells/mL of supplemented HL1 serum-free media were treated with 0.5 µM TAT peptide 48-57, TAT-Runx1.d190-K167A or TAT-Runx1.d190 protein for 4 hours. The cells were washed with PBS and lysed directly in 1x Laemmli sample buffer at a concentration of 1 x 10^6^ cells/10 µL buffer and incubated at 95^o^C for 5 minutes and on ice for 1 minute. The lysate was transferred to a QIAshredder column (Qiagen) and centrifuged at 13,000 rpm for 5 minutes at 4^o^C. Samples containing 1-4 x10^6^ cell equivalents were separated on a 10% SDS-PAGE and transferred to Immobilon P (Millipore) paper or Hybond-P PVDF (GE). The blot was probed with anti-c-Myc 9E10 antibody or goat polyclonal anti-actin (I-19) (Santa Cruz Biotechnology) as a loading control. Protein levels were determined by using Adobe Photoshop to quantify band intensities and background.

### Quantitative real-time PCR analysis

2 x 10^6^ Jurkat T cells or 6-8 x 10^6^ splenocytes were treated as described above and resuspended in 0.5 mL of Qiazol (Qiagen). RNA was extracted using an RNeasy MinElute kit (Qiagen), treated with DNAse (Invitrogen) and cDNA synthesis carried out using AMV Reverse Transcription System (Promega, Madison, WI) or iScript cDNA synthesis kit (Bio-Rad, Hercules, CA). Quantitative real-time PCR reactions were carried out using Takara SYBR Premix Ex Taq (Clontech) in the Mx3005P system (Agilent Technologies) according to the manufacturer’s instructions. The primers murine *GAPDH1* forward 5’-CCAATGTGTCCGTCGTGGATCTG-3’; murine *GAPDH1* reverse 5’-TGCCTGCTTCACCACCTTCT TG-3’; murine *c-Myc* forward 5’-GAGACACCGCCCACCACCAG-3’, murine *c-Myc* reverse 5’-AGCCCGACTCCGACCTCTTG-3’, were used with the cycling conditions: 95^°^C for 10 seconds, 95^°^C for 5 seconds, 60^°^C for 10 seconds, 72^°^C for 22 seconds for 40 cycles. The primers murine *GAPDH2* forward 5’-TCGTCCCGTAGACAAAATGG-3’; murine *GAPDH2* reverse, 5’-TTGAGGTCAATGAAGGGGTC-3’; murine *CD4* forward 5’-ACTGACCCTGAAGCAGGAGA-3’; murine *CD4* reverse, 5’-TCCTGGAGTCCATCTTGACC-3’, were used with the cycling conditions: 95^°^C for 10 seconds, 95^°^C for 5 seconds, 54^°^C for 10 seconds, 72 ^°^C for 22 seconds for a total of 40 cycles. *GAPDH2* primers were obtained from qprimerdepot and were used to generate Figure S2. The primers human *GAPDH* forward 5’- TGCACCACCAACT GCTTAGC-3’; human *GAPDH* reverse 5’-GGCATGGACTGTGGTCATGAG-3’; human *MYC* forward 5’-CAGCTGCTTAGACGCTGGATT-3’, human *MYC* reverse 5’-GTAGAAATACGGCTGCACCGA-3’; human *SERPINB2* forward 5’-GTTCATGCAGCAGATCCAGA-3’; human *SERPINB2* reverse 5’-CGCAGACTTCTCACCAAACA-3’ were used with the cycling conditions: 95^°^C for 10 seconds, 95^°^C for 5 seconds, 55^°^C for 10 seconds, 72^°^C for 22 seconds for 40 cycles. Human *GAPDH* and *MYC* primers were obtained from qprimerdepot. ΔC_τ_ was used to describe the difference between the threshold cycle (C_T_) value of the target gene and the C_T_ of the reference gene *GAPDH*, i.e., ΔC_τ =_ C_τ_ (target) -C_τ_(*GAPDH*). The relative mRNA expression level was calculated using the instrument’s software as 2^-[ΔC^τ^(treated cells) -ΔC^τ ^(untreated cells)]^. For randomly selected data sets, calculations were performed manually to confirm consistency with software results. PCR products were also resolved on a 2% agarose gel to ensure that only a single band of the correct size was present in samples.

### Statistical analysis

Statistical analysis was performed using Prism 5.0 software. Two-sided t tests with a 95% confidence interval were used to calculate any significant differences between 2 groups. P-values less than or equal to 0.05 were considered significant.

## Results

### Endogenous Runx1 occupies three consensus Runx-binding sites upstream of the human or murine *c-Myc* transcriptional start sites

**Figure 1:**
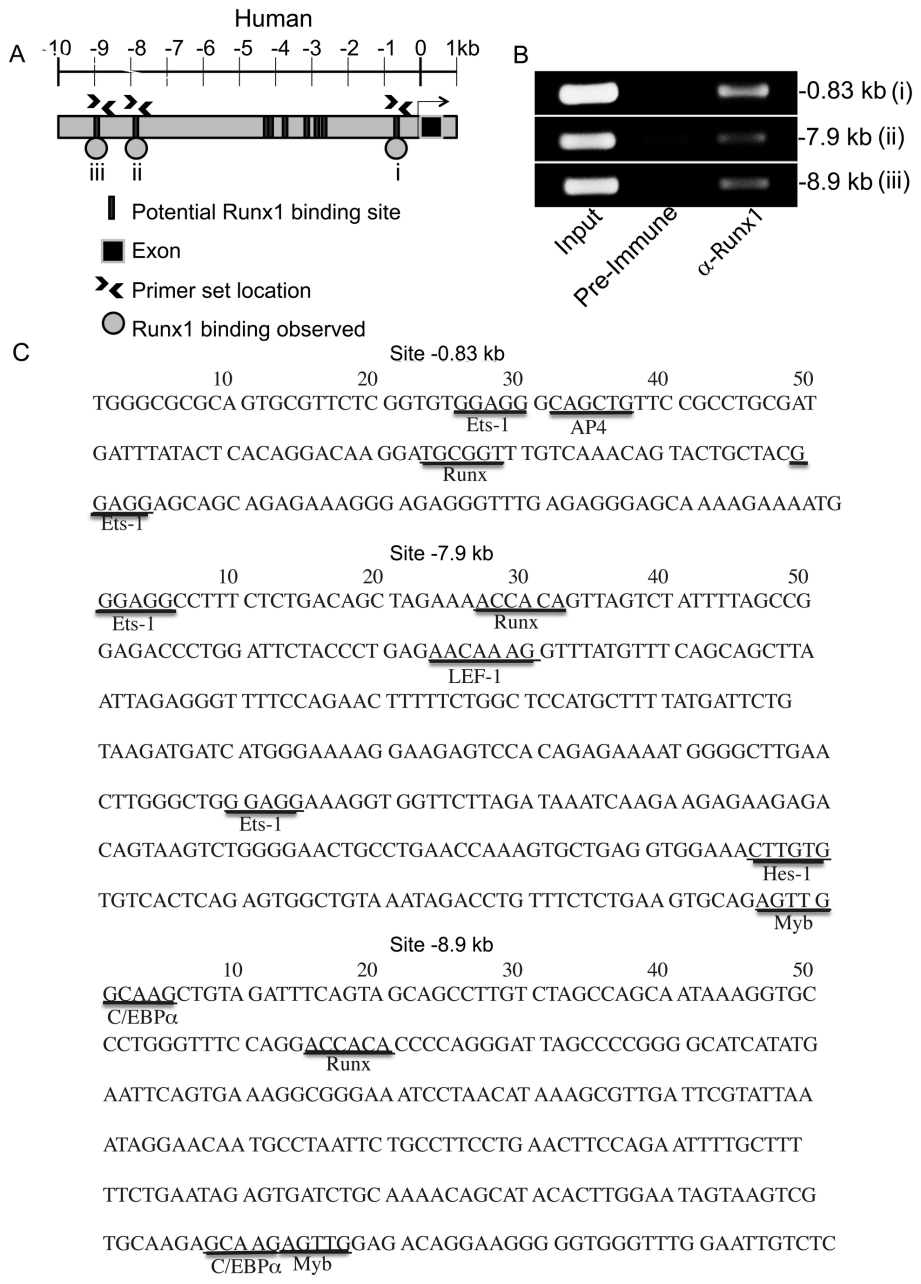
Endogenous Runx1 binds to multiple sites in the human *c-Myc* locus. (A) Schematic of the human *c-Myc* locus 10 kb upstream of the transcriptional start site, with consensus Runx binding sites (5’-TGCGGT-3’ or 5’-ACCACA-3’) indicated as grey rectangles. The locations of the PCR primers used in ChIP analysis are indicated by arrowheads and i, ii or iii. A grey circle indicates that endogenous Runx1 occupies that site. (B) ChIP analysis using human Jurkat T cells. A 1% fraction was reserved as an input control (Input) and the remaining chromatin was immunoprecipitated with preimmune sera (Pre-immune) or anti-distal Runx1 (α-Runx1). PCR was carried out using primer sets amplifying Runx binding sites at -0.83 (i), -7.9 (ii) and -8.9 kb (iii) upstream of the human *c-Myc* transcriptional start site. *N*=3. (C) Sequence surrounding consensus Runx binding sites. Binding sites for transcription factors known to functionally or physically interact with Runx transcription factors (Ets-1, Hes-1, PU.1, LEF-1, Myb and CEBPα [22,25,27,30,85,92]) or implicated in transcriptional silencing (AP4/ZEB E-box and RAP1 [86,87,93]) are identified [94].

**Figure 2:**
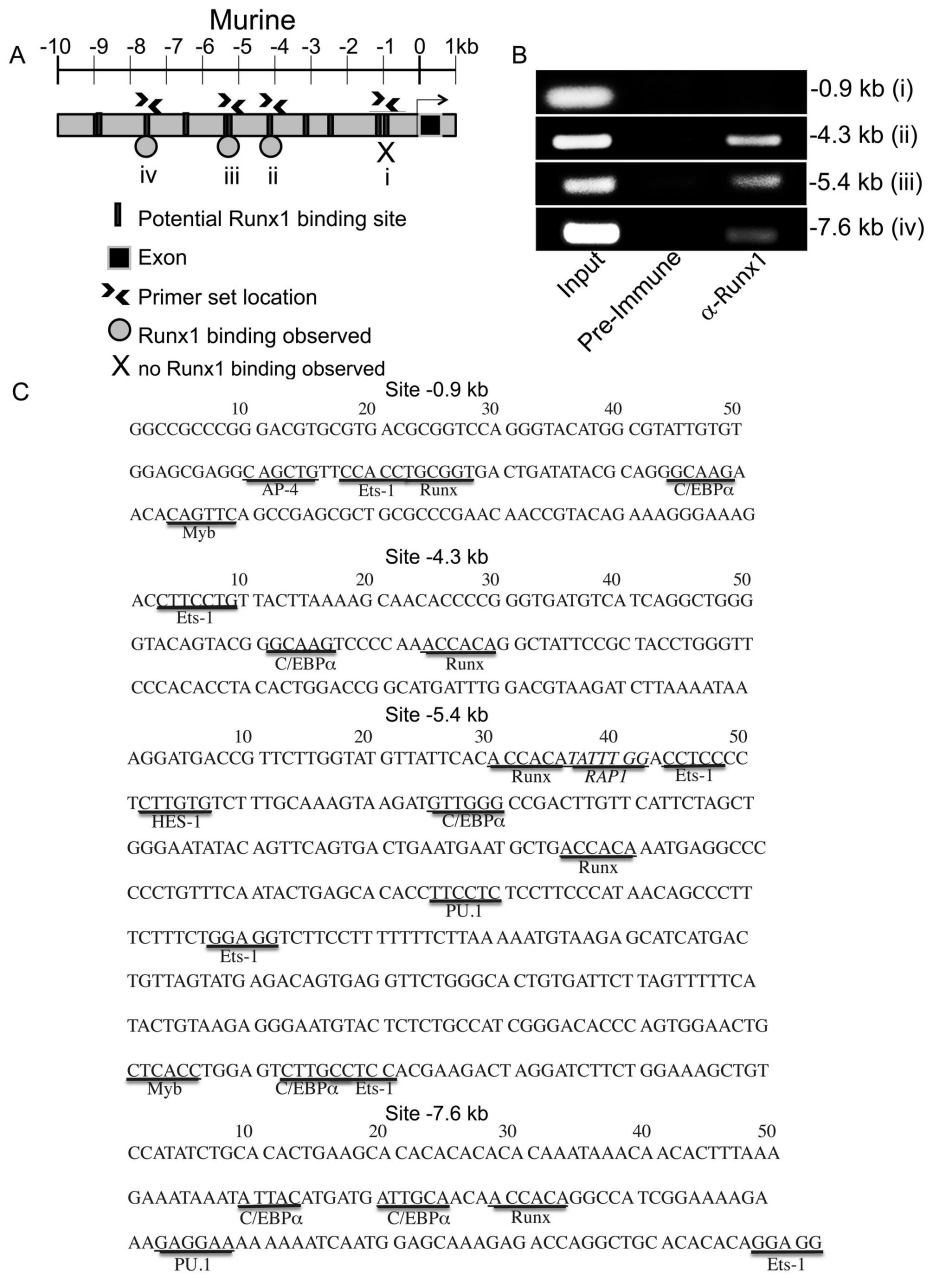
Endogenous Runx1 binds to multiple sites in the murine *c-Myc* locus. (A) Schematic of the murine (A) *c-Myc* locus 10 kb upstream of the transcriptional start site with consensus Runx binding sites indicated as grey rectangles. The locations of the PCR primers used in ChIP analysis (shown in B) are indicated by arrowheads and i, ii, iii or iv. An X indicates a Runx binding site that is not occupied by endogenous Runx1; a grey circle indicates that endogenous Runx1 occupies that site. (B) ChIP analysis using primary murine splenocytes. A 1% fraction was reserved as an input control (Input) and the remaining chromatin was immunoprecipitated with preimmune sera (Pre-immune) or anti-distal Runx1 (α-Runx1). PCR was carried out using primer sets amplifying (B) Runx binding sites located at -0.9 (i), -4.3 (ii), -5.4 (iii) and -7.6 kb (iv) upstream of the murine *c-Myc* transcriptional start site. *N*=3. (C) Sequence surrounding consensus Runx binding sites. Binding sites for transcription factors known to collaborate with Runx transcription factors (Ets-1, Hes-1, PU.1, LEF-1, Myb and CEBPα [22,25,27,30,85,92]) or implicated in transcriptional silencing (AP4/ZEB E-box and RAP1 [86,87,93]) are identified [94].

In order to determine whether Runx1 is capable of regulating c-Myc expression, we carried out chromatin immunoprecipitation analysis (ChIP) to characterize Runx1 binding to the *c-Myc* locus. We surveyed the *c-Myc* locus 10 kb upstream of the start site in humans (Figure 1A) and mice (Figure 2A) for potential consensus Runx binding sites. There are nine to eleven consensus Runx binding sites in the human and murine 10 kb regions, for which we designed seven primer sets. Three of these primer sets (arrow heads, Figures 1A and 2A) generated a PCR product of the correct size from input chromatin and chromatin precipitated by an anti-Runx1 antibody recognizing the first 19 amino acids of distal Runx1, but not from chromatin precipitated by the negative control preimmune sera. These results indicated that endogenous distal Runx1 binds to multiple sites in the *c-Myc* locus in human Jurkat T cells (Figure 1B) and murine primary splenocytes (Figure 2B). Several other primer sets did not generate a PCR product of the correct size or amplified a product from chromatin precipitated by the negative control preimmune sera and were thus ineligible for further use. However, a primer set amplifying a PCR product of the correct size containing the Runx-binding site at position -0.9 kb from input murine splenocyte chromatin did not amplify the equivalent PCR product from chromatin precipitated by either the negative control preimmune sera or by the anti-Runx1 antibody, indicating that endogenous distal Runx1 does not bind to the consensus Runx-binding site closest to the murine *c-Myc* transcriptional start site (Figure 2A, 2B).

### Runx1 lacking its C-terminal half upregulates human *c-Myc* transcription

**Figure 3:**
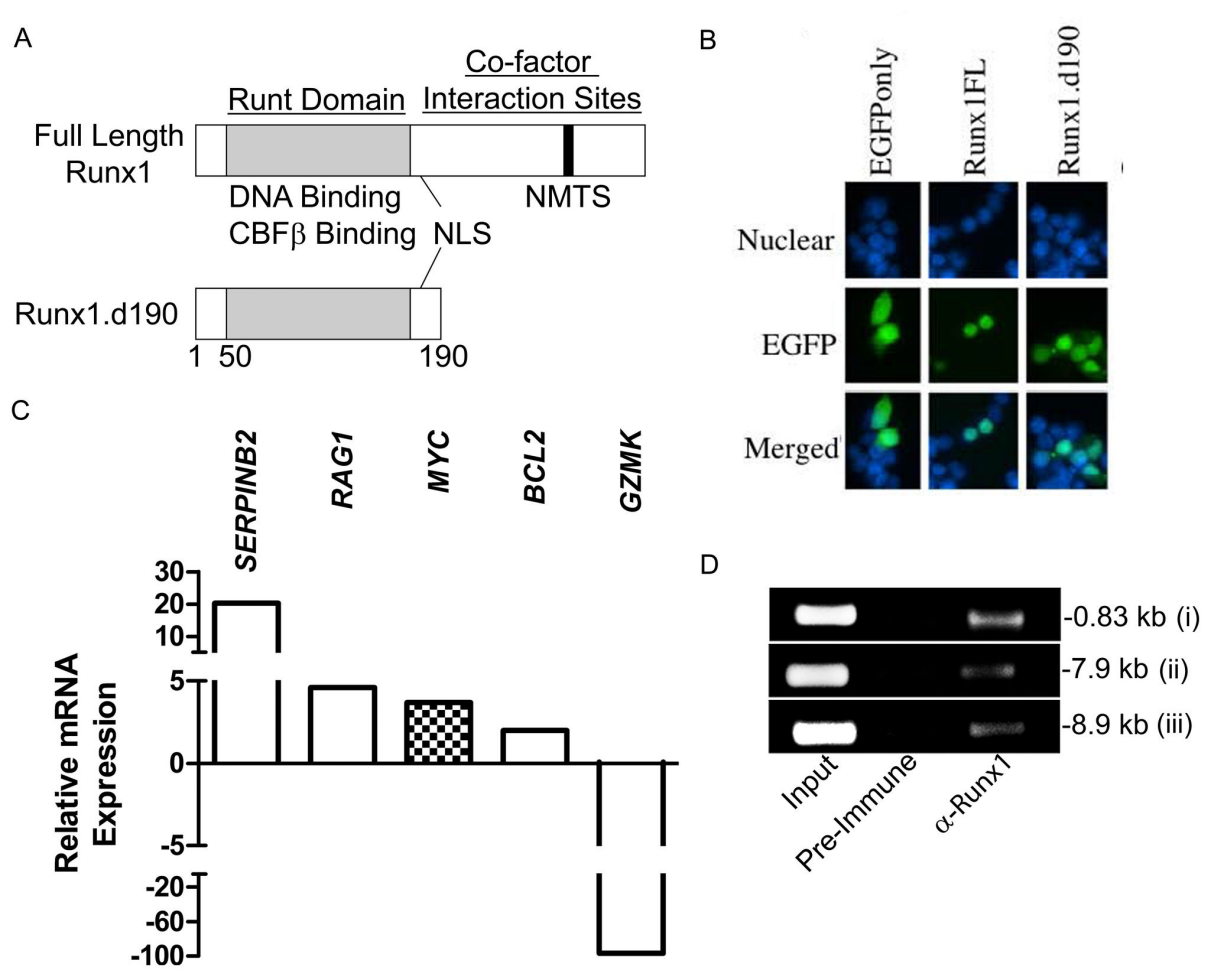
Human Jurkat T cells lentivirally transduced with Runx1.d190 show increased transcription of *c-Myc*. (A) Schematic of the structure of Runx1 and Runx1.d190. (B) 293T. cells were transfected with empty pEGFP-N1 vector (EGFPonly, left column) as a control for cytoplasmic staining, pEGFP-N1 vector containing full-length Runx1 fused in-frame to EGFP (Runx1FL, middle column) or Runx1.d190 fused in-frame to EGFP (Runx1.d190, right column). The nuclear DNA was visualized by staining with Hoescht 33342 (Nuclear, top row). Nuclear (top row) and EGFP (middle row) fluorescence are shown in isolation and merged (Merged, bottom row). (C) Relative differences in transcription between Jurkat T cells lentivirally transduced with control empty vector or vector encoding Runx1.d190 as determined by microarray analysis are shown. A complete listing of genes whose transcription is affected by Runx1.d190 in Jurkat T cells is located at http://www.ncbi.nlm.nih.gov/geo/. (D) ChIP analysis. Chromatin was prepared from Jurkat T cells lentivirally transduced with Runx1.d190 and immunoprecipitated with preimmune sera (Pre-immune) or anti-distal Runx1 (α-Runx1). PCR was carried out using primer sets amplifying Runx1-binding sites at -0.83 (i), -7.9 (ii) and -8.9 kb (iii) upstream of the human *c-Myc* transcriptional start site. *N*=3.

**Figure 4:**
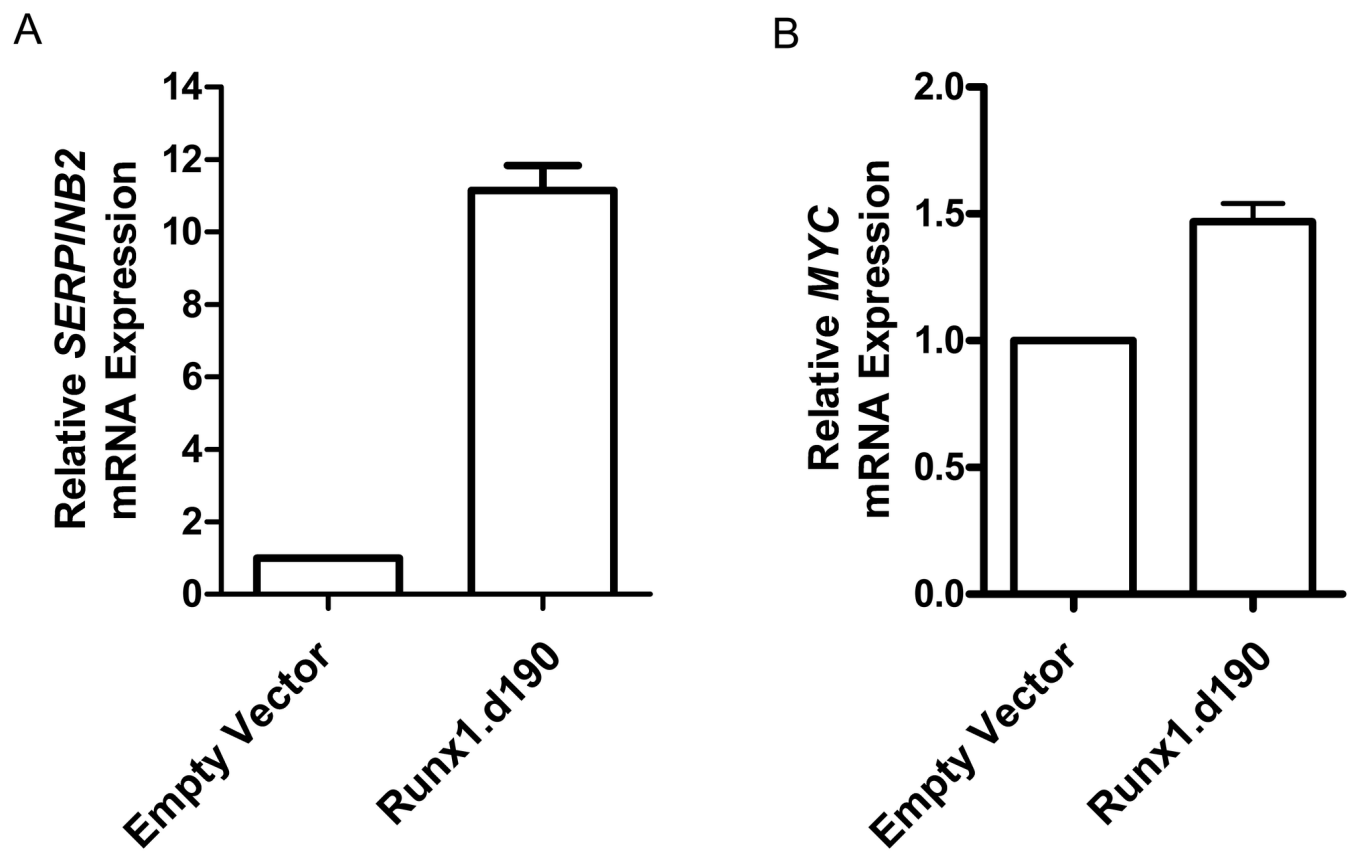
Real-time PCR confirms human Jurkat T cells retrovirally transduced with Runx1.d190 exhibit increased transcription of *SERPINB2* and *c-Myc*. Relative differences in transcription for *SERPINB2* (A) and *MYC* expression (B) between Jurkat T cells retrovirally transduced with control empty vector or vector encoding Runx1.d190 (Runx1.d190) as determined by quantitative RT-PCR are shown. Columns represent the mean of data from two independent experiments in which duplicates were analyzed. Error bars represent standard deviation from the mean.

We next examined whether and in what direction Runx transcription factors regulated *c-Myc* transcription in human Jurkat T cells lentivirally transduced with a C-terminally truncated form of Runx1 (Runx1.d190). Runx1.d190 retains the runt DNA-binding domain and nuclear localization sequence but lacks the C-terminal half of the protein, which contains important cofactor interaction sites (Figure 3A). Like full-length Runx1, Runx1.d190 preferentially localizes to the nucleus of cells (Figure 3B). Additionally, Runx1.d190 binds to DNA more efficiently than full-length Runx1 and exhibits activity opposite to that of Runx transcription factors for C-terminal dependent functions [36,37]. Thus, we predicted that lentivirally-transduced Runx1.d190 would act in dominant opposition to endogenous Runx1 present in the control Jurkat T cells transduced with empty vector. We carried out microarray analysis using Illumina BeadArrays to characterize the effect of Runx1.d190 on *c-Myc* transcription as well as other targets of Runx1. Microarray analyses showed that several important genes exhibit either positive or negative changes in their expression upon transduction with Runx1.d190, which is consistent with the context-dependent repressive or transactivating activity of Runx transcription factors. The transcript level of *c-Myc* is increased in Runx1.d190-transduced Jurkat cells compared to the empty pLEIGW vector control in microarray and quantitative real-time PCR analysis, implying that endogenous full-length Runx1 normally represses c-Myc expression (Figures 3C, 4B). The serpin protease inhibitor B2 (*SERPINB2*), which has not been previously shown to be a target of Runx, is also upregulated in microarray and quantitative real-time PCR analysis (Figures 3C, 4A). Recombination activating gene 1 (RAG1) [38] and the anti-apoptotic gene *BCL2* [75], which are known to be repressed by full-length Runx1, are also upregulated, which is consistent with the dominant inhibitor nature of Runx1.d190 (Figure 3C). In contrast, the cytolytic serine protease granzyme K (*GZMK*) is downregulated, which is consistent with studies that show that Runx family transcription factors transactivate the granzyme family member granzyme B [76] (Figure 3C). There are ten consensus Runx-binding sites less than 10 kb upstream of the *SERPINB2* start site and the regulatory region 10 kb upstream of the *GZMK* start site contains nine consensus Runx-binding sites (data not shown), suggesting that Runx transcription factors bind directly to both loci. ChIP analysis of human Jurkat T cells transduced with Runx1.d190 shows Runx occupancy of the human *c-Myc* locus at positions i, ii, and iii (Figure 3D). Although the anti-distal Runx1 antisera used in the ChIP analysis recognizes both endogenous Runx1 (Figures 1B, 2B, and 3D) and the lentivirally transduced Runx1.d190 (Figure 3D), the change in the transcription of *c-Myc* and other genes upon expression of Runx1.d190 on a background of endogenous Runx1 suggests that Runx1.d190 is occupying at least one of the three Runx-binding sites examined upstream of the human *c-Myc* transcriptional start site.

### Membrane permeable C-terminally truncated Runx1 binds to the murine *c-Myc* enhancer and upregulates transcription of *c-Myc*

**Figure 5:**
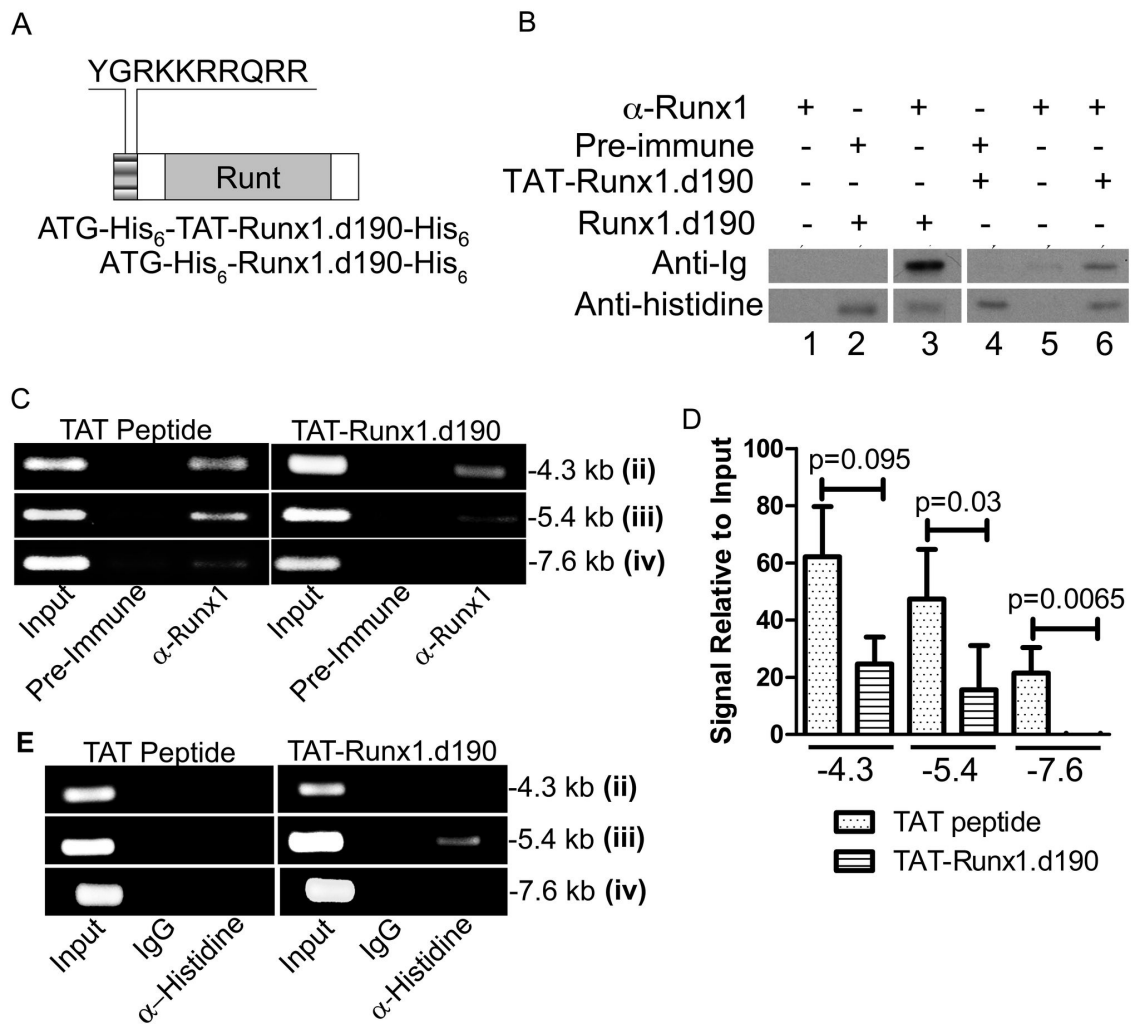
Binding of membrane-permeable TAT-Runx1.d190 protein to the murine *c-Myc* locus. (A) Schematic of TAT-Runx1.d190 fusion protein. The Runx1.d190 protein lacks the TAT peptide, but has N-terminal and C-terminal polyhistidine tags. (B) Differential affinity of polyclonal antisera recognizing the N-terminus of distal Runx1 for Runx1.d190 and TAT-Runx1.d190 proteins. Histidine-tagged Runx1.d190 (lanes 2 and 3), or TAT-Runx1.d190 protein (lanes 4 and 6) adhered to nickel beads, or nickel beads alone (lanes 1 and 5) were fixed in 1% formaldeyde to simulate ChIP conditions and incubated with control Pre-immune sera (lanes 2 and 4) or anti-distal Runx1 (α-Runx1) antisera (lanes 1, 3, 5 and 6). The amount of pre-immune or anti-distal Runx1 immunoglobin associated with fixed Runx1.d190 or TAT-Runx1.d190 proteins is shown on a representative immunoblot probed with anti-immunoglobin (Anti-Ig). The amount of Runx1.d190 or TAT-Runx1.d190 proteins immobilized on the beads prior to fixation is shown on a representative immunoblot probed with anti-polyhistidine (Anti-histidine). *N*=3. (C) ChIP analysis of chromatin from murine splenocytes treated with 0.5 µM TAT peptide or TAT-Runx1.d190 protein and immunoprecipitated with preimmune sera (Pre-immune) or anti-distal Runx1 (α-Runx1). PCR was carried out with primer pairs amplifying consensus Runx-binding sites at -4.3 (ii), -5.4 (iii), and -7.6 (iv) kb upstream of the murine *c-Myc* transcriptional start site. A representative of three independent experiments is shown. (D) The PCR product yields from chromatin from TAT peptide- or TAT-Runx1.d190-treated murine splenocytes immunoprecipitated with anti-distal Runx1, relative to input, are graphed. The PCR primers used are indicated on the x-axis. P-values derived from a two-tailed t test indicating statistical significance are shown above the brackets. (E) ChIP analysis of chromatin prepared from murine splenocytes treated with 0.5 µM TAT peptide or TAT-Runx1.d190 protein and immunoprecipitated with control IgG (IgG) or anti-polyhistidine (α-Histidine). PCR was carried out with primer pairs amplifying consensus Runx-binding sites at -4.3 (ii), -5.4 (iii), and -7.6 (iv) kb upstream of the murine *c-Myc* transcriptional start site. *N*=3.

Jurkat cells are an immortalized T cell leukemia cell line, with potential dysregulation in multiple oncogenes. Lentiviral integration into the genome and transcriptional regulation of the inserted gene adds to the variability of this system. To more precisely determine the temporal nature of expression of *c-Myc* in response to Runx1.d190 in normal cells, we treated murine primary splenocytes with an early and transiently acting cell membrane-permeable fusion protein consisting of Runx1.d190 and the cell penetrating peptide TAT (Figure 5A). The TAT peptide is capable of transporting proteins to which it is fused across cell membranes rapidly, with efficiencies close to 100%, and is thus an excellent tool for delivering Runx1.d190 into primary cells [77–80]. TAT-Runx1.d190 was purified under denaturing conditions in order to increase its membrane-translocation efficiency (Figure S1). TAT-Runx1.d190 labeled with fluorescein isothiocyanate (FITC) was visualized inside non-adherent human leukemic NK cells within 15 minutes (Figure S2A). TAT-Runx1.d190 protein is active as demonstrated by the derepression of *CD4* (Figure S2B) which is a known function of Runx1.d190 [36]. Attempts to generate functional TAT-Runx1 were unsuccessful (data not shown). The TAT-Runx1.d190 construct consists of Runx1.d190 fused to the TAT cell penetrating peptide (YGRKKRRQRR) at its N-terminus, along with both N-terminal and C-terminal polyhistidine tags (Figure 5A). The anti-distal Runx1 antisera recognizes epitopes in the N-terminal 19 amino acids of Runx1 transcribed from the distal P1 promoter. In order to determine whether the fusion of the TAT sequence to the N-terminus of the Runx.d190 affects its recognition by the anti-distal Runx1 antisera, we examined the binding of anti-distal Runx1 antisera to equivalent amounts of TAT-Runx1.d190 and Runx1.d190 immobilized on beads. Both proteins have polyhistidine tags at their N and C-termini, differing only in the presence of the TAT peptide fused to the N-terminus of TAT-Runx1.d190 (Figure 5A). Less anti-distal Runx1 antisera is bound by TAT-Runx1.d190 (Figure 5B, lane 6) than by an equivalent amount of Runx1.d190 (Figure 5B, lane 3), predicting that the anti-distal Runx1 antisera will preferentially recognize endogenous distal Runx1 in ChIP experiments. To determine whether TAT-Runx1.d190 could compete with endogenous Runx1 for binding to Runx consensus sites in the *c-Myc* locus, primary murine splenocytes were treated with TAT peptide (control) or TAT-Runx1.d190 after which ChIP analysis was carried out using anti-distal Runx1 antibody. Compared to the TAT peptide treatment control, treatment with TAT-Runx1.d190 fusion protein significantly decreased the association of distal Runx1 recognized by the anti-distal Runx1 antisera at all three sites examined (Figure 5C, D). The decrease in immunoprecipitated distal Runx1 binding to the c-Myc locus upon TAT-Runx1.d190 treatment is consistent with TAT-Runx1.d190 displacing endogenous distal Runx1. To determine whether TAT-Runx1.d190 replaced endogenous Runx1 at any of these sites, ChIP analysis was carried out using an anti-polyhistidine antibody specifically recognizing only the histidine-tagged TAT-Runx1.d190 protein. TAT-Runx1.d190 replaced endogenous Runx1 on the murine *c-Myc* locus at the -5.4 kb site (Figure 5E). Although TAT-Runx1.d190 association with the -4.3 kb and -7.6 kb sites was not detectable with the anti-polyhistidine antibody, this could be attributable to the presence of two Runx binding sites within 100 bp at -5.4 kb versus only one Runx binding site each at -4.3 kb and -7.6 kb (Figure 2C). The increased density of TAT-Runx1.d190 at -5.4 kb site may have increased the avidity of the anti-polyhistidine antibody for TAT-Runx1.d190-DNA complexes so that the immunoprecipitated DNA could be detected. Alternatively, TAT-Runx1.d190 treatment may have indirectly blocked endogenous Runx transcription factors from binding to the -4.3 kB and -7.6 kb sites through catalyzing nucleosome movement.

### The effect of cell membrane permeable TAT-Runx1.190 on murine *c-Myc* transcription is dependent on the continued binding of TAT-Runx1.190 to the locus

**Figure 6:**
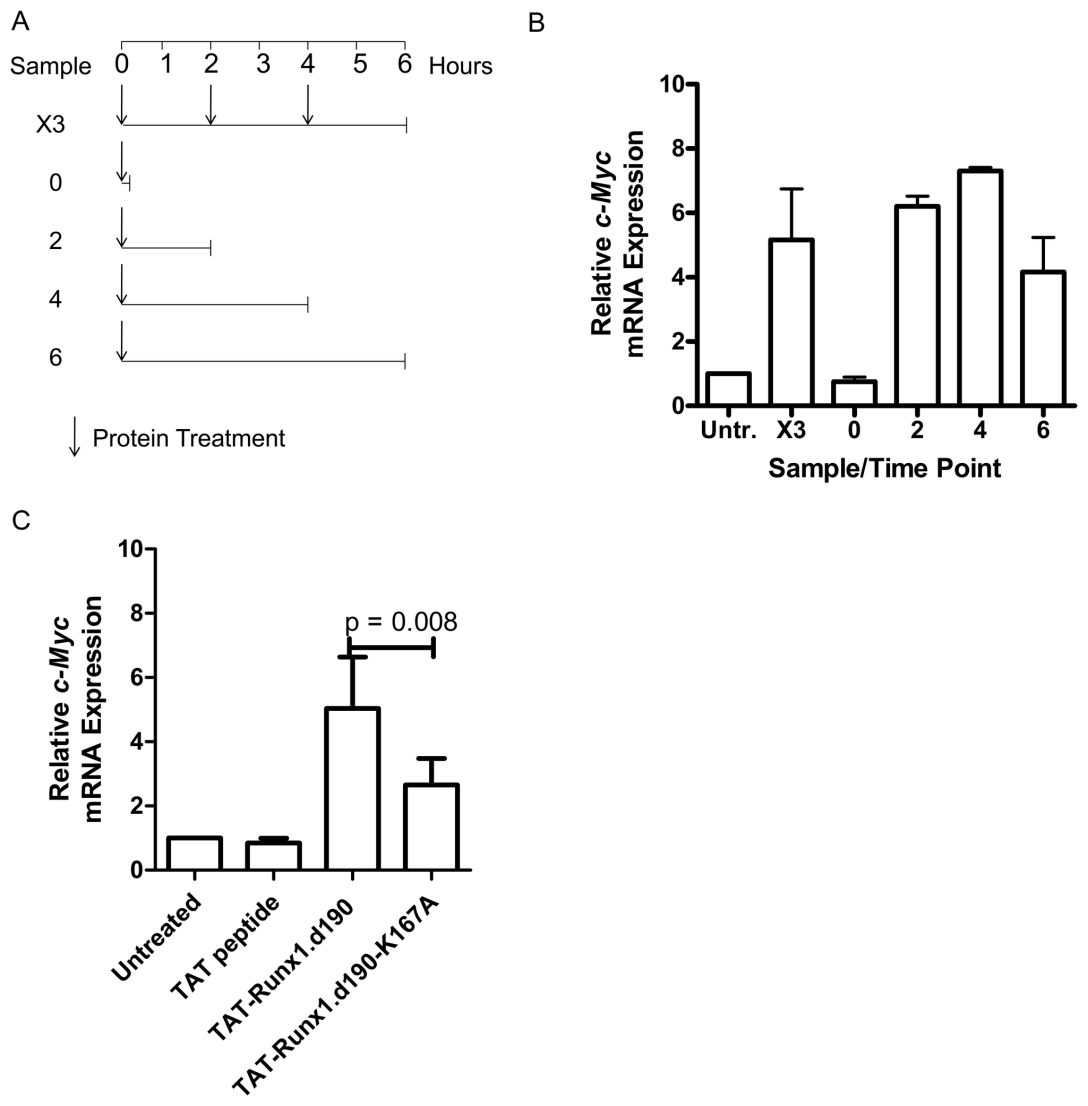
TAT-mediated Runx1.d190 increases *c-Myc* transcription in primary murine splenocytes. (A) Experimental design. Murine splenocytes were left untreated (Untr.), or treated with 0.5 µM TAT-Runx1.d190 protein every 2 hours for a total of 3 treatments (X3), and harvested at the 6 hour time point. Alternatively, splenocytes were given a single protein treatment of 0.5 µM TAT-Runx1.d190 protein and harvested at 0, 2, 4 or 6 hours post-treatment. (B) SYBR Green real time PCR was carried out using cDNA prepared from the samples treated as in (A). The results of 2 independent experiments are shown. Treatment conditions described in (A) are indicated on the x-axis. Bars represent standard deviation from the mean. (C) Real-time PCR was carried out using cDNA from murine splenocytes treated with 0.5 µM TAT peptide, TAT-Runx1.d190, or TAT-Runx1.d190-K167A for 4 hours. The results of three independent experiments are shown. Bars represent standard deviation from the mean. A p-value indicating statistical significance derived from a two-tailed t test is shown.

In order to investigate the timing of transactivation of *c-Myc* by TAT-Runx.d190, we treated primary murine splenocytes with 0.5 µM TAT-Runx1.d190 every 2 hours, harvesting 2 hours after the third treatment (sample X3, Figure 6A). Alternatively, cells were given a single treatment of 0.5 µM TAT-Runx1.d190 at 0 hours and harvested immediately, or at 2, 4, or 6 hours post-treatment (Figure 6A). The transcript level of *c-Myc* is significantly increased in response to either single or repeated TAT-Runx1.d190 protein treatments (Figure 6B). It is interesting to note that there is no significant difference in the increase in transcript levels in response to three treatments and harvest at 6 hours compared to only one treatment of TAT-Runx1.d190 and harvest 6 hours later, indicating that the effect of the initial dose of recombinant TAT-Runx1.d190 is maximal and stable for at least 6 hours (Figure 6B). *C-Myc* transcript levels return to baseline in splenocytes harvested 24 hours after one treatment with TAT-Runx1.d190 (data not shown), suggesting that TAT-Runx1.d190 does not catalyze a long-lasting epigenetic modification of the *c-Myc* locus.

To investigate whether the increase in *c-Myc* expression is dependent on TAT-Runx1.d190 binding to Runx binding sites and the ensuing blockage of endogenous Runx1 binding, we treated splenocytes with TAT-Runx1.d190 containing a mutation in the Runx1 runt DNA-binding domain (K167A), which has previously been shown to decrease the binding of Runx1 to DNA [43,45,47]. Splenocytes treated with TAT-Runx1.d190-K167A protein exhibited a significant decrease in *c-Myc* transcription compared to the mRNA levels observed in splenocytes treated with wildtype TAT-Runx1.d190 protein (Figure 6C), which is consistent with a model in which TAT-Runx1.d190 is displacing endogenous Runx transcription factors by binding to Runx binding sites in the DNA and inducing the transcription of *c-Myc* by acting in opposition to endogenous Runx1 with an intact C-terminus.

### The increase in murine *c-Myc* transcription mediated by TAT-Runx1.d190 leads to an increase in c-Myc protein

**Figure 7:**
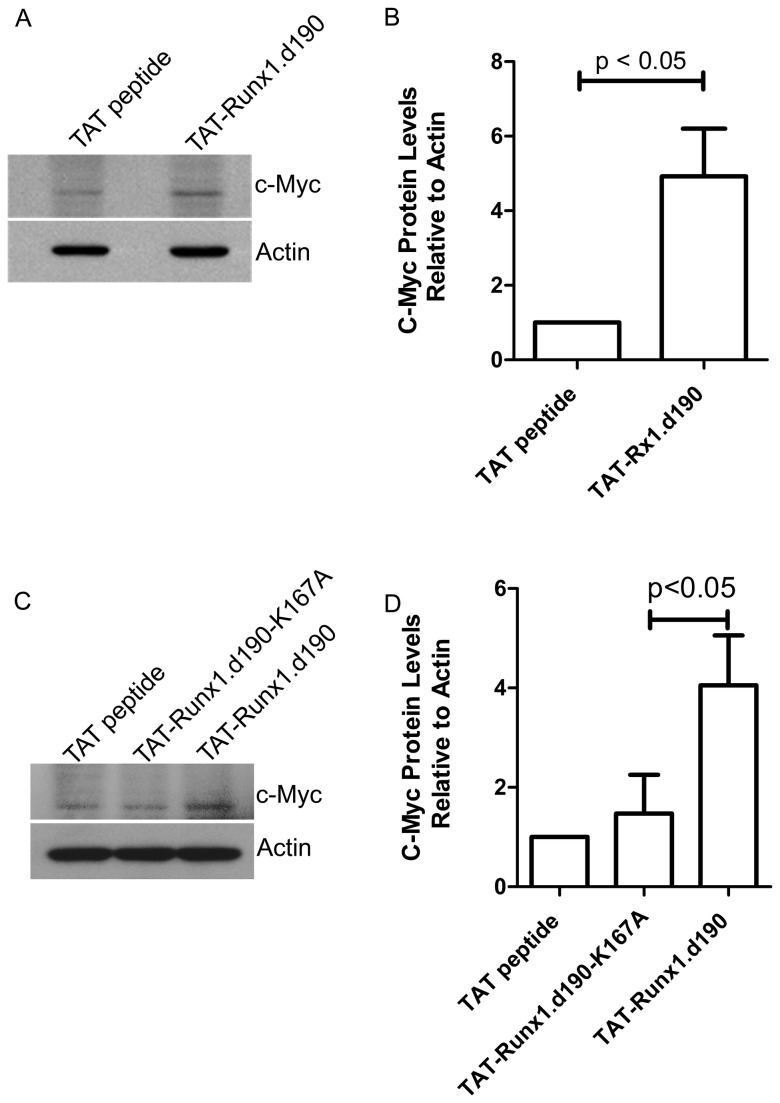
Increased *c-Myc* mRNA transcription correlates with increased c-Myc protein levels. Murine splenocytes were treated with 0.5 µM TAT peptide, TAT-Runx1.d190-K167A or TAT-Runx1.d190 for 4 hours. (A, B) Representative immunoblots of whole cell lysates probed with c-Myc 9E10 antibody (top panel) or a goat polyclonal anti-actin antibody (bottom panel) are shown. (B, D) Quantification of c-Myc protein levels normalized to actin levels from immunoblots. Bars represent standard deviation from the mean. A p-value indicating statistical significance derived from a two-tailed t test is shown. *N*=4 (A), *N*=3 (B).

We next examined whether the increase in *c-Myc* transcription induced by TAT-Runx1.d190 treatment leads to an increase in c-Myc protein. Primary murine splenocytes were treated with 0.5 µM TAT-Runx1.d190, TAT-Runx1.d190-K167A protein or TAT peptide as a control for 4 hours. Whole cell lysates were immunoblotted with c-Myc 9E10 antibody or a goat polyclonal anti-actin antibody as a loading control. C-Myc protein levels in cells treated with TAT-Runx1.d190 were significantly increased compared to those in cells treated with TAT peptide control (Figure 7A,B) or with the DNA binding mutant TAT-Runx1.d190-K167A (Figure 7B), which is consistent with an increase in *c-Myc* transcripts resulting in a rapid increase in c-Myc protein.

## Discussion

Runx1 directly regulates the transcription of *c-Myc* in hematopoietic cells in a C-terminal-dependent and DNA-binding-dependent manner. Endogenous Runx1 occupies at least three sites on the human or murine *c-Myc* locus. It is likely that endogenous Runx1 and the other two Runx family transcription factors repress *c-Myc* transcription, since the introduction of a C-terminally deleted form of Runx1 (Runx1.d190) that acts in opposition to full-length Runx1 or Runx3 on the CD4 silencer results in the increased transactivation of *c-Myc* [36]. This effect is seen with microarray and quantitative real-time PCR analyses of lentivirally transduced human Jurkat T cells or with primary murine cells treated with a membrane-permeable form of Runx1.d190, indicating that the Runx-mediated repression of *c-Myc* transcription is evolutionarily conserved. The rapid increase in *c-Myc* transcription seen after treatment with the Runx1.d190 membrane-permeable protein, coupled with the occupancy of the *c-Myc* locus by either endogenous Runx1 or by TAT-Runx1.d190, supports the hypothesis that this is a direct effect on the *c-Myc* locus. The upregulation of c-Myc transcripts and protein by the C-terminally deleted form of Runx1 is important in the context of Runx1 mutations and translocations that remove the C-terminus and are associated with cancerous transformation; full-length Runx transcription factors may serve as tumor suppressors that tune down the expression of oncogenic c-Myc.

Runx1.d190 also upregulates transcription of the serine protease inhibitor serpinB2/plasminogen activator inhibitor-2 gene (*SERPINB2*) and downregulates transcription of granzyme K (*GZMK*). It is likely that full-length Runx transcription factors downregulates *SERPINB2* and upregulates *GZMK* expression directly as both loci contain multiple consensus Runx binding sites upstream of the transcriptional start site. At least one member of the serpin family has been shown to inhibit one of the granzyme genes; thereby protecting cancer cells from cytolysis (reviewed in 81], and [82). Additionally, high levels of *GZMK* released from NK cells have been shown to play a protective role in multiple sclerosis by killing activated T cells associated with CNS inflammation [83]. Thus, full-length Runx transcription factors may act on various gene targets including c-Myc with the aggregate affect of suppressing cancerous transformation.

Runx transcription factors can act as cell context-dependent transactivators or repressors. It is thought that they do so by recruiting other transcription factors or chromatin-modifying enzymes, via interactions with the Runx C-terminus and DNA-binding domain. One explanation for the effect of Runx1.d190 on *c-Myc* transcription is that Runx1.d190 acts by displacing endogenous Runx1 on critical *c-Myc* locus Runx binding sites and that its lack of a C-terminus prevents it from interacting with important repressive co-factors, such as AP4, ZEB, Hes-1, Myb RAP1, TLE/Groucho, Ear-2, Suv39H1 histone methyltransferase, or histone deactylases, or localizing appropriately in the nucleus through its nuclear matrix targeting sequence (NMTS) (reviewed in 84). The best-characterized Runx-controlled silencers are an intronic sequence in the *CD4* locus and an intergenic silencer located between RAG-1 and RAG-2 [36,38,85–89]. These silencers control the expression of genes whose expression are tightly developmentally regulated and permanently silenced in many cell populations; however both *CD4* and *c-Myc* transcription is more dynamically regulated. It remains to be seen whether all Runx binding sites are required to be occupied for Runx repression of c-Myc or whether other transcription factors are required to make Runx binding sites accessible for binding. Hes-1, Myb and the E-box-binding bHLH transcription factors AP4 and ZEB bind to the CD4 silencer and collaborate with Runx transcription factors to repress CD4 transcription [85–88]. Both AP4 and Runx1 repress p21 transcription [90,91]. The same collaborations may be taking place on the c-Myc locus, as there are E-boxes and Myb and Hes-1 binding sites upstream of the c-Myc transcriptional start site in both the human and murine c-Myc loci (Figures 1C, 2C). AP4 is itself induced by c-Myc, which suggests a possible negative feedback loop controlling c-Myc expression [90].

It is of interest that the increase in *c-Myc* transcription caused by a single treatment of TAT-Runx1.d190 at 6 hours pre-harvest is not significantly different than the increase attributable to multiple treatments with TAT-Runx1.d190 at 6, 4 and 2 hours pre-harvest. This suggests that the first dose of protein saturates Runx1-binding sites and that the protein is stable on the DNA for approximately 24 hours post-treatment, which is when c-Myc transcription returns to baseline (data not shown). DNA binding by TAT-Runx1.d190 is required, in that a single point mutation that decreases DNA-binding (K167A) results in a protein that neither significantly increases *c-Myc* transactivation nor c-Myc protein. The K167A Runx1 mutant used in our studies shows some binding in the presence of its non-DNA-binding subunit CBFβ, which is ubiquitously expressed [45,47]. It is likely that the slight increase in *c-Myc* transcripts and protein seen in cells treated with TAT-Runx1.d190K167A is due to this phenomenon.

C-Myc is a powerful transcription factor at the nexus of normal cell processes and oncogenesis. Thus, the regulation of *c-Myc* transcription by Runx transcription factors has significant implications for our understanding of how Runx family transcription factors regulate hematopoiesis and oncogenesis.

## Supporting Information

Figure S1 Purification of a 25 kDa TAT-Runx1.d190 protein under denaturing conditions. (A) Representative Coomassie stained SDS-PAGE gel. The bacterial lysate (lane 1) containing the protein was incubated overnight with nickel beads under 8M urea denaturing conditions. The flow-thru supernatant (lane 2) containing unbound proteins was removed before the nickel beads were washed (lane 3) extensively to remove non-specifically bound proteins. The TAT-Runx1.d190 protein was eluted from the washed beads using 200 and 500 mM imidazole (lanes 4 and 5). The protein was concentrated (lane 6) and further purified using a PD-10 desalting column (lanes 7 and 8) to exchange remaining urea/imidazole buffer for PBS containing 10% glycerol. Any remaining LPS was removed by polymyxin beads leaving a relatively pure final fraction (lane 9). An arrowhead indicates TAT-Runx1.d190 protein. (B) Representative immunoblot of the fractions described in (A) probed with anti-polyhistidine antibody.
(TIF)

Figure S2 Characterization of TAT-Runx1.d190 fusion protein activity. (A) Detection of fluorescein isothiocyanate (FITC)-labeled TAT-Runx1.d190 association with non-adherent human leukemic NK YT cells. YT cells were incubated with 1.2 µM FITC-labeled Runx1.d190 or BSA for 10 minutes at 37^o^C. The cells were washed extensively and analyzed by flow cytometry. FITC-labeled TAT-Runx1.d190 and FITC-labeled BSA treated cell populations are indicated, with FITC fluorescent intensity on the x-axis. (B) TAT-Runx1.d190 represses *CD4* expression. Thymocytes from Eμ-Bcl-2-25 mice were incubated with media only (untreated), or treated with 0.2 µM BSA, TAT peptide, or TAT-Runx1.d190 for 4 hours (4^°^C for the first 30 minutes followed by 37^°^C for 3.5 hours). Bars represent standard deviation from the mean. *N*=3.
(TIF)
