# Supplementary figures and images for: Runx Transcription Factors Repress Human and Murine c-Myc Expression in a DNA-Binding and C-Terminally Dependent Manner
Source: PLoS One. 2013 Jul 18;8(7):e69083. doi: 10.1371/journal.pone.0069083 (PMC3715461; doi:10.1371/journal.pone.0069083)

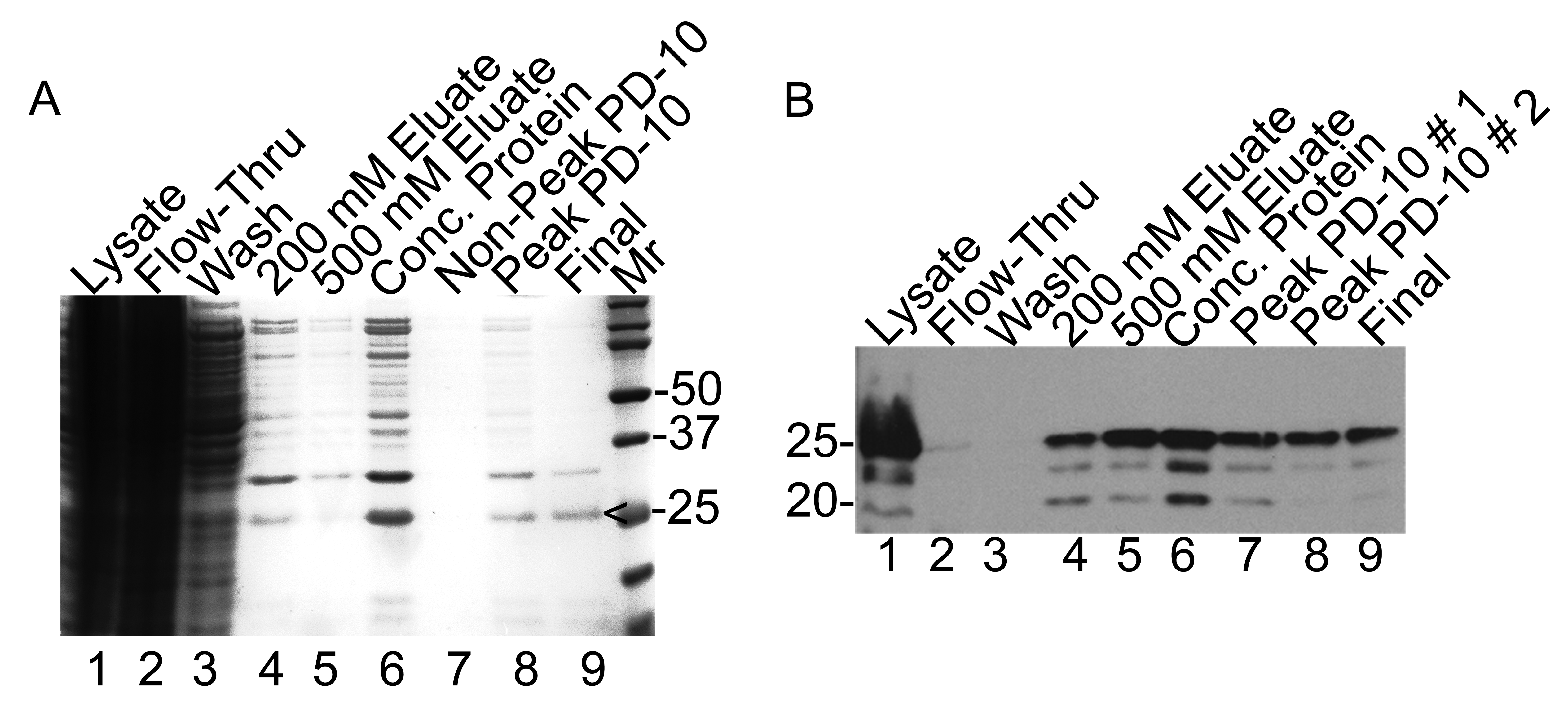

Supplement: Figure S1 — (A) Representative Coomassie stained SDS-PAGE gel. The bacterial lysate (lane 1) containing the protein was incubated overnight with nickel beads under 8M urea denaturing conditions. The flow-thru supernatant (lane 2) containing unbound proteins was removed before the nickel beads were washed (lane 3) extensively to remove non-specifically bound proteins. The TAT-Runx1.d190 protein was eluted from the washed beads using 200 and 500 mM imidazole (lanes 4 and 5). The protein was concentrated (lane 6) and further purified using a PD-10 desalting column (lanes 7 and 8) to exchange remaining urea/imidazole buffer for PBS containing 10% glycerol. Any remaining LPS was removed by polymyxin beads leaving a relatively pure final fraction (lane 9). An arrowhead indicates TAT-Runx1.d190 protein. (B) Representative immunoblot of the fractions described in (A) probed with anti-polyhistidine antibody. (TIF) [file pone.0069083.s001.tif]

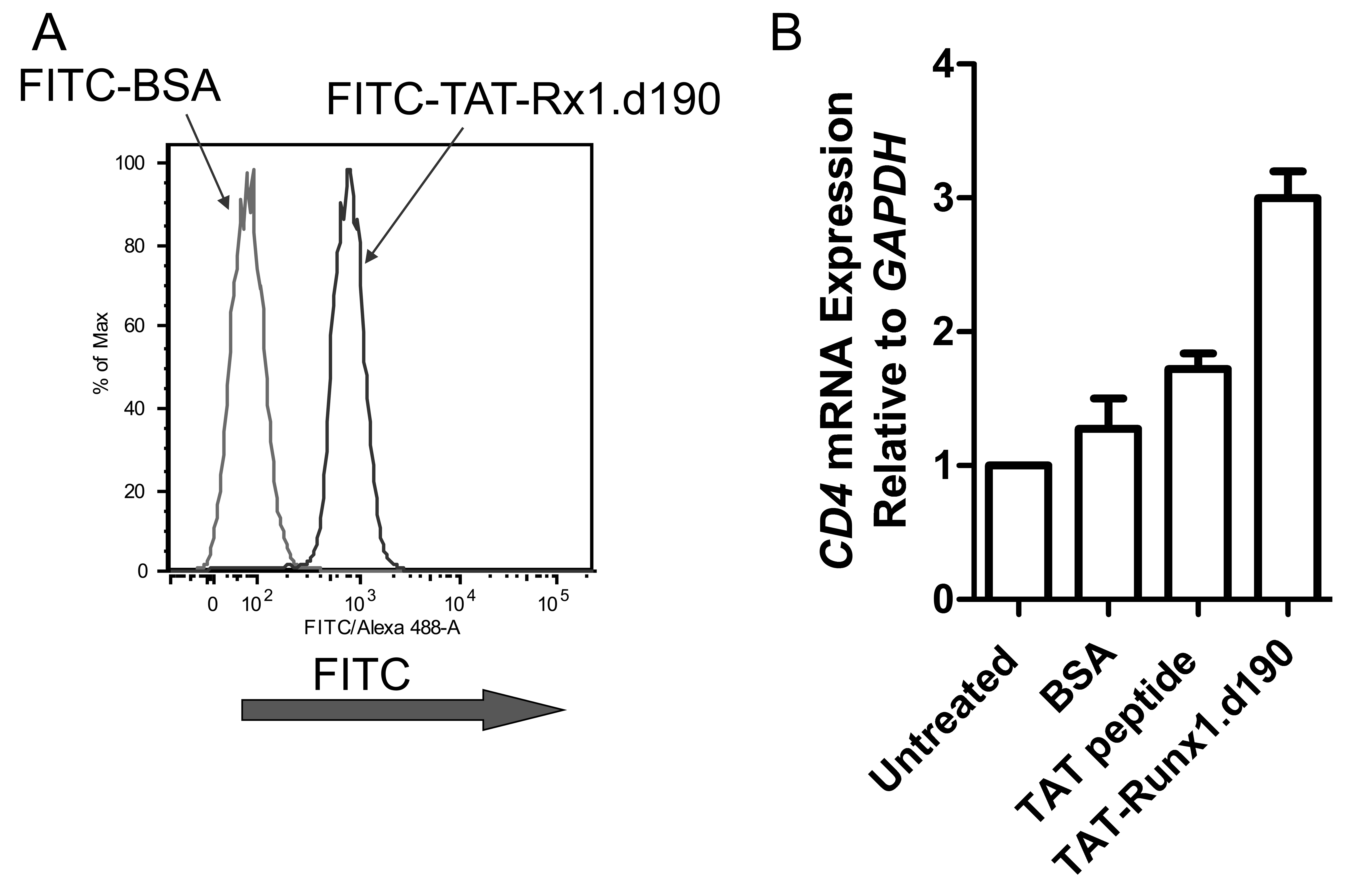

Supplement: Figure S2 — (A) Detection of fluorescein isothiocyanate (FITC)-labeled TAT-Runx1.d190 association with non-adherent human leukemic NK YT cells. YT cells were incubated with 1.2 µM FITC-labeled Runx1.d190 or BSA for 10 minutes at 37oC. The cells were washed extensively and analyzed by flow cytometry. FITC-labeled TAT-Runx1.d190 and FITC-labeled BSA treated cell populations are indicated, with FITC fluorescent intensity on the x-axis. (B) TAT-Runx1.d190 represses CD4 expression. Thymocytes from Eμ-Bcl-2-25 mice were incubated with media only (untreated), or treated with 0.2 µM BSA, TAT peptide, or TAT-Runx1.d190 for 4 hours (4°C for the first 30 minutes followed by 37°C for 3.5 hours). Bars represent standard deviation from the mean. N=3. (TIF) [file pone.0069083.s002.tif]
